# Limited susceptibility of rhesus macaques to a cowpox virus isolated from a lethal outbreak among New World monkeys

**DOI:** 10.5194/pb-4-163-2017

**Published:** 2017-09-11

**Authors:** Kerstin Mätz-Rensing, Constanze Yue, Jeanette Klenner, Heinz Ellerbrok, Christiane Stahl-Hennig

**Affiliations:** 1 German Primate Center, Göttingen, Germany; 2 Robert Koch Institute, Highly Pathogenic Viruses (ZBS 1), Berlin, Germany; a present address: Paul Ehrlich Institute, Frankfurt, Germany

## Abstract

This study was undertaken to investigate the susceptibility of rhesus monkeys to the calpox virus, an orthopoxvirus (OPXV) of the *Cowpox virus* species (CPXV), which is uniformly lethal in common marmosets. Six rhesus monkeys were either intravenously (i.v.) or intranasally (i.n.) exposed to the virus. Monitoring of the macaques after viral exposure included physical examinations, the determination of viral load by real-time PCR and plaque assay, and the analysis of humoral responses. Two i.v. inoculated animals developed numerous classical pox lesions that started after inoculation at days 7 and 10. Both animals became viremic and seroconverted. They exhibited maximal numbers of lesions of approximately 50 and 140 by day 21. One animal completely recovered, while the other one suffered from a phlegmonous inflammation of a leg initially induced by a secondarily infected pox lesion and was euthanized for animal welfare reasons. In contrast to previous pathogenicity studies with the calpox virus in marmosets, none of the four animals inoculated intranasally with doses of the calpox virus exceeding those used in marmosets by orders of magnitude showed typical clinical symptoms. No viral DNA was detectable in the blood of those animals, but three animals seroconverted. In two of these three animals, infectious virus was sporadically isolated from saliva. This indicates that rhesus monkeys are less susceptible to calpox virus infection, which limits their use in further intervention studies with OPXV.

## 1 Introduction

The genus *Orthopoxvirus* (OPXV) comprises several species including *Variola virus* (VARV), *Monkeypox virus* (MPXV), *Cowpox virus* (CPXV) and *Vaccinia virus* (VACV) that are all, more or less, pathogenic for humans. Fortunately, the causative agent of smallpox, VARV, has successfully been eradicated by a worldwide vaccination campaign led by the World Health Organization (WHO) (Fenner, 1988; World Health Organization, 1980). On the recommendation of the WHO, the vaccination program was stopped in the 1980s because of severe side effects of the vaccine. The number of people lacking immunity against smallpox and other zoonotic OPXV infections is therefore increasing (Shchelkunov, 2013). Consequently, a potential biowarfare attack with smallpox would hit a nearly unprotected population. In addition, the zoonotic potential of orthopoxviruses, e.g., the increasing incidence of human monkeypox in the Democratic Republic of the Congo (Rimoin et al., 2010), is of a growing concern. The 2003 outbreak in the USA caused by prairie dogs infected by imported exotic pets from Africa clearly showed that monkeypox is not restricted to Africa and can be transmitted at any time to any place (Centers for Disease, C. a. P., 2003; Reed et al., 2004). So far, no licensed antiviral treatment for poxvirus infections is available. There are two vaccines with irregular disposability. In the USA the second generation vaccine ACAM2000^®^ has been licensed since 2007 (http://www.fda.gov/ohrms/dockets/ac/07/briefing/2007-4292b2-02.pdf), and Imvanex^®^, based on the modified vaccinia virus Ankara, received a marketing authorization by the European Commission in 2013. Nevertheless, in light of increasing numbers of zoonotic infections with different orthopoxviruses new vaccines and therapeutic agents are urgently needed. Both have to be tested in adequate animal models. Since small animal models have considerable limitations regarding disease pathology and pharmacokinetics, animal models with nonhuman primates (NHPs), the closest relatives to humans, are essential.

Over the last decades several animal models for OPXV were developed in NHPs (Schmitt et al., 2014). Unfortunately, none of the animal models fulfills all the criteria needed and all have limitations (Hutson and Damon, 2010; Safronetz et al., 2013). Based on the Food and Drug Administration animal efficacy rule, new drugs and vaccines must be tested in more than one species and one of the species should be a nonhuman primate animal model (Snoy, 2010).

The current nonhuman primate model for smallpox is the intravenous (i.v.) inoculation of MPXV, which causes a fulminant disease with many similarities to that of the human disease. A great disadvantage of the intravenous infection route is that it does not mimic the natural route of smallpox transmission, which occurs through close contact or inhaled aerosols. Key events of a natural pox infection such as the alteration of the upper respiratory tract, a primary viremic phase and prodromal phases are skipped. Nevertheless, these models cause a systemic disease with mortality rates of up to 100 % and can be used to evaluate the efficacy of anti-OPXV therapeutics (Huggins et al., 2009) and vaccines (summarized in Schmitt et al., 2014).

To mimic the natural route of infection, respiratory models using MPXV, which often causes fibrinonecrotic bronchopneumonia that resembles the human MPXV and smallpox disease course, were developed (Goff et al., 2011; Johnson et al., 2011a; Zaucha et al., 2001). However, the work with this animal model is complicated because of safety restrictions. The experiments must be performed in a biosafety level (BSL)-3 containment facility. Furthermore, the VARV animal model, a suitable model for hemorrhagic smallpox used in some drug efficacy studies (Huggins et al., 2009; Mucker et al., 2013) as well as for pathogenesis studies (Wahl-Jensen et al., 2011), is restricted to two BSL-4 laboratories worldwide (Centers for Disease Control and Prevention, USA; and State Research Center of Virology and Biotechnology, Russia). Thus, alternative models are needed, and those based on CPXV, which is classified as a BSL-2 pathogen, are increasing in interest.

CPXV has the broadest host range of all OPXV. Compared to MPXV or VARV, research with CPXV can be done under median safety conditions. Cynomolgus macaques have been infected intravenously and intrabronchially with CPXV to study pathogenesis (Johnson et al., 2011b; Smith et al., 2012). Small-particle aerosol inoculation in rhesus macaques resulted in a severe respiratory disease (Johnson et al., 2015). Recently, we developed a nonhuman primate model based on marmosets experimentally infected with the calpox virus that belongs to the *Cowpox virus* species (Kramski et al., 2010). Common marmosets are highly susceptible to the calpox virus and they can be infected experimentally via an intravenous or intranasal (i.n.) route (Mätz-Rensing et al., 2006, 2012). The intranasal route of infection resembles the natural infection route of smallpox and is therefore a suitable model for the validation of therapeutics and vaccines. A disadvantage of this model is that some species-specific reagents for analyses are not available yet. The investigation of the immune and the inflammatory response is especially impeded as there is little knowledge on inflammatory markers in this species. In contrast, a wide range of commercial systems to investigate the inflammatory response and the cytokine and chemokine profile in the rhesus monkey model is available.

We therefore wanted to investigate whether the fulminant disease of marmosets following infection with the calpox virus as described above can be reproduced in rhesus monkeys. If successful this could represent an alternative model allowing for extended functional immunological studies on orthopoxvirus pathogenesis. Based on the assumption that older rhesus monkeys might be more susceptible to calpox infection because of waning immune competence, we inoculated aged monkeys with the calpox virus by the same routes as described for marmosets (Kramski et al., 2010). Our results showed that, compared to marmosets, this species was not only less susceptible to infection but, depending on the route of inoculation, hardly developed severe clinical disease.

**Table 1:** Overview of inoculation route and clinical, pathohistological and virological findings.*

Animal	Age	Inoculation	Clinical findings	Pathohistologic	Viral	Development of
number	(years)	route,		findings	DNA in	of OPXV-
		volume,			blood	specific
		and dose			and/or	antibodies
					tissue	
1	19	i.v., 1 mL,	severe poxvirus-	severe	yes	yes
		7 × 10${}^{5}$ PFU	induced skin	pyogranulomatous		
			alterations	dermatitis,		
				*Pneumonyssus*		
				pneumonia		
2	23	i.v., 1 mL,	poxvirus-	*Pneumonyssus*	yes	yes
		3.5 × 10${}^{6}$ PFU	induced skin	pneumonia,		
			alterations	leiomyoma of the		
			which healed	uterus		
3	21	i.n., 180 µL,	three single	*Pneumonyssus*	no	no
		7 × 10${}^{5}$ PFU	poxvirus-	pneumonia		
			induced lesions			
			within the			
			pharynx and			
			one at the			
			corner of one			
			eye			
4	22	i.n., 180 µL,	no poxvirus-	*Pneumonyssus*	no	yes
		7 × 10${}^{5}$ PFU	induced	pneumonia,		
			alterations	leiomyoma of the		
				uterus		
5	20	i.n., 180 µL,	no poxvirus-	endometriosis,	no	yes
		7 × 10${}^{5}$ PFU	induced	leiomyoma of the		
			alterations	uterus		
6	23	i.n., 180 µL	no poxvirus-	endometriosis,	no	yes
		7 × 10${}^{5}$ PFU	induced	leiomyoma of the		
			alterations	uterus		

${}^{*}$ i.v., intravenous; i.n., intranasal; PFU, plaque forming units.

## 2 Materials and methods

### 2.1 Animals

Six healthy sexually mature female rhesus monkeys (*Macaca mulatta*) belonging to the age category “aged macaques” (Asquith et al., 2012) were obtained from the breeding colony of the German Primate Center (see Table 1). The age was between 19 and 23 years. Older monkeys were chosen assuming that they were more susceptible to orthopoxvirus infection than younger ones as reported for humans (Fenner et al., 1989). During the experiments the animals were housed in single cages with visual, olfactory and acoustic contact to one another. They were allowed free access to food and water and provided standard environmental enrichment. All animals were adequately fed and cared for in accordance with the German Animal Welfare act. The animal experiments were approved by the responsible veterinary authorities (approval number: 33.14-42502-04-095/09) and performed in accordance with the EU guidelines for the accommodation and care of animals used for experimental and other scientific purposes. Animals were clinically checked twice a day. Blood samples were collected on days 4, 7, 10, 14, 17, 21 and at necropsy. Animals 2–6 were also sampled on day 24 and 28. Animals that were moribund or terminally ill were humanely euthanized using an overdose of barbiturates. Severe apathy, weight loss, and inflammation of the upper respiratory tract in combination with pox-like alterations on skin and mucous membranes were the criteria used to define terminally ill animals. Only animal 1 was euthanized for animal welfare reasons at day 21 after infection. To conclude the study, all remaining animals were euthanized 10 weeks after viral exposure. Complete post-mortem examinations were performed on all animals. A comprehensive organ spectrum was collected for histological, electron microscopical and virological analyses at necropsy.

### 2.2 Viral strain and application route

The calpox virus stock used for inoculation has been described before (Kramski et al., 2010). The viral titer was determined by plaque assay prior to its use in infection experiments. The virus stock contained 3.5 × 10${}^{6}$ PFU (plaque forming units) mL${}^{-1}$.

The rhesus monkeys were infected intravenously with two different infectious doses of the calpox virus (Table 1), depending on the virus titer, the volume per ampoule (200 µL) and overall availability of the stock. The first i.v. dose corresponded to 200 µL of undiluted virus. In order to potentially augment clinical symptoms, the dose for the second i.v. inoculated animal was increased fivefold. Since whether intranasal inoculation would also lead to infection of rhesus monkeys like in marmosets was unpredictable, we chose undiluted virus for this route. For the purpose of virus inoculation, monkeys were anesthetized by injecting 0.1 mL per 1 kg body weight of a mixture containing 5 % ketamine, 1 % xylazine and 0.01 % atropine into the hamstring muscles (*Musculus semimembranosus*, *Musculus semitendinosus*, *Musculus biceps femoris*). A volume of 1 mL calpox virus was administered intravenously into the great saphenous vein. For intranasal inoculation, three doses of 60 µL of undiluted virus suspension were alternatingly applied into the nostrils.

### 2.3 Isolation of total DNA from tissues and whole blood, determination of viral genome equivalents and infectious particles in saliva

Extraction of total DNA from tissue was performed in a FastPrep apparatus (MP Biomedicals, Illkirch, France) with two intervals of 20 s at 6 m s${}^{-1}$ for tissue homogenization as described by Kramski et al. (2010). Nucleic acid was extracted from lysate using the DNeasy Blood and Tissue Kit (Qiagen, Hilden, Germany). DNA from whole blood was extracted using the DNA Blood Mini Kit (Qiagen, Hilden, Germany). Extractions were performed according to the manufacturer's instructions, and DNA was eluted in 100 µL AE buffer (Qiagen). Quantification of viral genomes in purified DNA from tissue or blood was amplified with a calpox-virus-specific qPCR assay as reported (Kramski et al., 2010). Quantities of viral DNA were expressed as genome equivalents (GE) per mL of blood or for tissue samples per 10${}^{6}$ copies of the c-*myc* gene. To investigate oropharyngeal virus shedding, we analyzed saliva for the presence of infectious virus by a plaque assay (Kramski et al., 2010).

### 2.4 Determination of binding and neutralizing antibodies

For the detection of binding antibodies an indirect ELISA was performed (Miller et al., 2011), except that ELISA plates were coated with 400 ng virus lysate per well prepared from the calpox-virus-infected Hep2 cells by sonication and subsequent centrifugation (5 min at 200 g). Plasma samples were applied in a single 1 : 400 dilution, and for detection of binding a HRP-coupled goat anti-human IgG antibody (Invitrogen, Karlsruhe, Germany) was used. Neutralizing antibodies were determined by plaque reduction neutralization test (PRNT) and the titer was calculated (Kramski et al., 2010).

### 2.5 Histological examination

Samples for histopathology were immersion fixed in 10 % neutral buffered formalin. Tissues underwent routine histological processing and immunohistological investigation as previously published (Mätz-Rensing et al., 2012). Additionally, selected sections of skin samples were processed for transmission electron microscopy (TEM) after glutaraldehyde fixation (2.5 %) and embedding in Epon.

**Figure 1:**
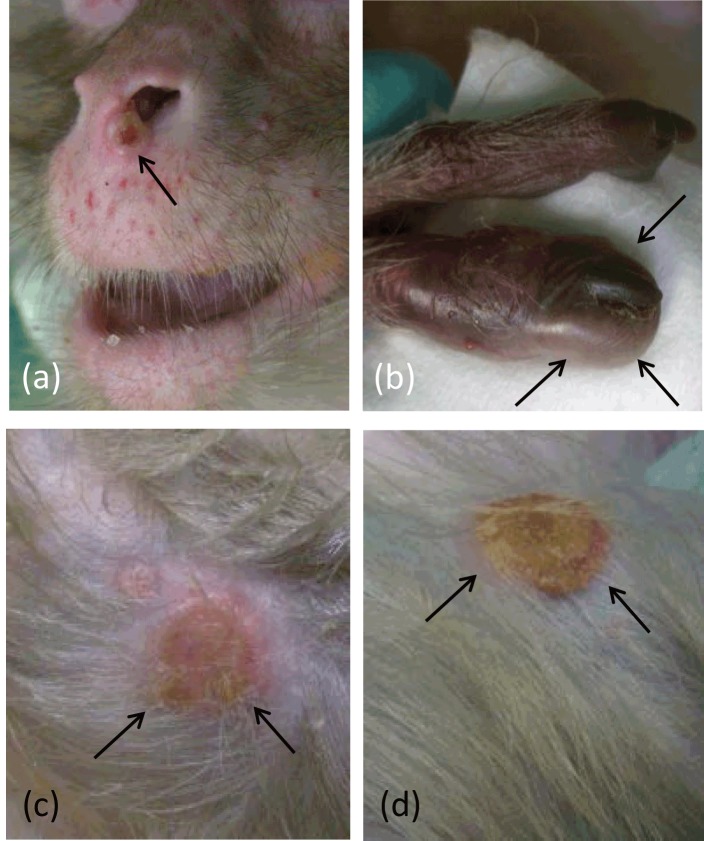
Dermal lesions at different time points after i.v. calpox infection, *Macaca mulatta*, animal no. 2. **(a)** Focal umbilicated pustular lesion beneath the nose, 17 days p.i. (arrows). **(b)** Severe focal papular dermatitis of the left trigger finger, 17 days p.i. (arrows). **(c)** Severe focal ulcerative dermatitis of the right arm induced by confluent pustules, 17 days p.i. (arrows). **(d)** Severe focal ulcerative dermatitis of the right arm covered by a crust, 21 days p.i. (arrows).

## 3 Results

### 3.1 Intravenous inoculation leading to vesiculopustular rash

Two animals were inoculated by the intravenous route. Numerous classical pox lesions started to develop on day 7 (animal 1) and 10 (animal 2) post-inoculation. The first clinical symptoms in both animals were exanthemas of the face and the upper parts of the extremities as well as small single macular lesions that appeared on the face, trunk and legs. Macular lesions spread over the whole body and began to develop into small papular and vesicular lesions. On day 10 and 12, pustules were spread over the entire body and began to umbilicate. In addition, lesions occurred on oral and genital mucous membranes and the mucocutaneous junctions. The lesions were completely umbilicated between day 14 and 17 (Fig. 1a). Moreover, the palms of hands and soles of feet were affected. Here, umbilication of the lesions was not observed (Fig. 1b). Lesions on the extremities tended to coalesce (Fig. 1c). The maximal number and size of lesions was observed at day 21 (approximately 50 for animal 1 and 140 for animal 2). In animal 1 the lesions reached diameters of up to 3 cm, were highly confluent and became necrotic, leading to a massive phlegmon of the right leg. This animal was immediately euthanized for animal welfare reasons at day 21 post-infection (p.i.). In animal 2 the pox lesions reached a maximal size of 1 cm in diameter, started to crust, and became dry and flattened (Fig. 1d). Scabs developed and fell off after about 4 weeks. After 5 weeks all lesions were completely healed.

**Figure 2:**
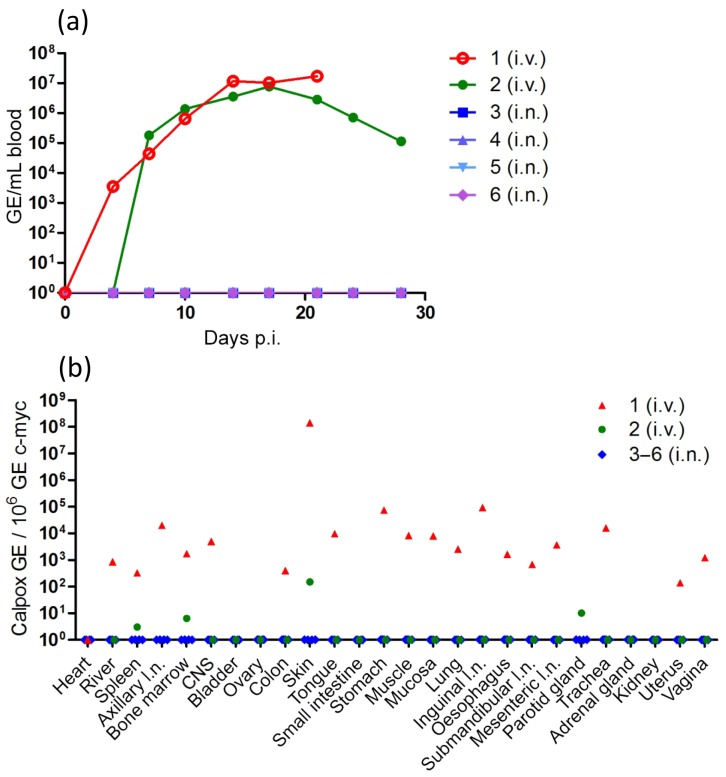
Detection of calpox DNA in blood and tissues of infected animals. **(a)** At different time points post-infection (see materials and methods), blood samples were taken, DNA was prepared from whole blood and viral DNA was determined with a specific qPCR assay. Genome equivalents (GE) for viral DNA were calculated per mL of blood. **(b)** Viral load in tissue. Post-mortem samples from various tissues were collected and DNA was isolated. Viral genome equivalents were determined by q-PCR and correlated to 10${}^{6}$ copies of the c-*myc* gene.

#### 3.1.1 Viral load after intravenous inoculation

After intravenous infection, calpox virus DNA was first detectable in blood between day 4 (animal 1) and 7 (animal 2). In animal 1 viral DNA continuously increased until day 14 and reached a plateau with a maximum of 1.7 × 10${}^{7}$ GE per mL blood on day 21, just prior to its euthanization for animal welfare reasons. In animal 2 the viral load peaked at day 17 with 7.7 × 10${}^{6}$ GE per mL blood and subsequently gradually decreased by almost 2 orders of magnitude until the analysis was stopped (Fig. 2a). Quantification of viral DNA in various tissues on the day of necropsy revealed that in animal 1 (day 21 p.i.) calpox virus was present in most of the investigated tissues (Fig. 2b). The highest viral load of 1.4 × 10${}^{8}$ GE per 1 × 10${}^{6}$ copies of c-*myc* was observed in the skin of animal 1. Lower viral loads in the range of 1.4 × 10${}^{2}$ to 9.3 × 10${}^{4}$ GE were measured in lymphatic, intestinal, mucosal, muscular and nervous tissues; in the liver; and in the respiratory and reproductive organs. No viral DNA was found in the heart, kidneys, adrenal glands, bladder, small intestine, parotid gland or ovaries. In animal 2 (day 70 p.i. and after recovery) calpox virus PCR was clearly positive in skin only (1.5 × 10${}^{2}$ GE), with low-level detection in the parotid gland, bone marrow and spleen. The detection of high copy numbers of viral DNA in many tissues of animal 1 at the time of sacrifice as well as residual viral DNA in some of the tissues in animal 2 after recovery indicated a systemic infection of the rhesus monkeys infected by the i.v. route (Fig. 2b). Saliva for the analysis of infectious virus was available from animals 2–6. In animal 2 all samples collected between day 4 and 24 were positive in the plaque assay, indicating the presence of infectious virus in saliva (Table 2).

#### 3.1.2 Humoral immune responses

**Figure 3:**
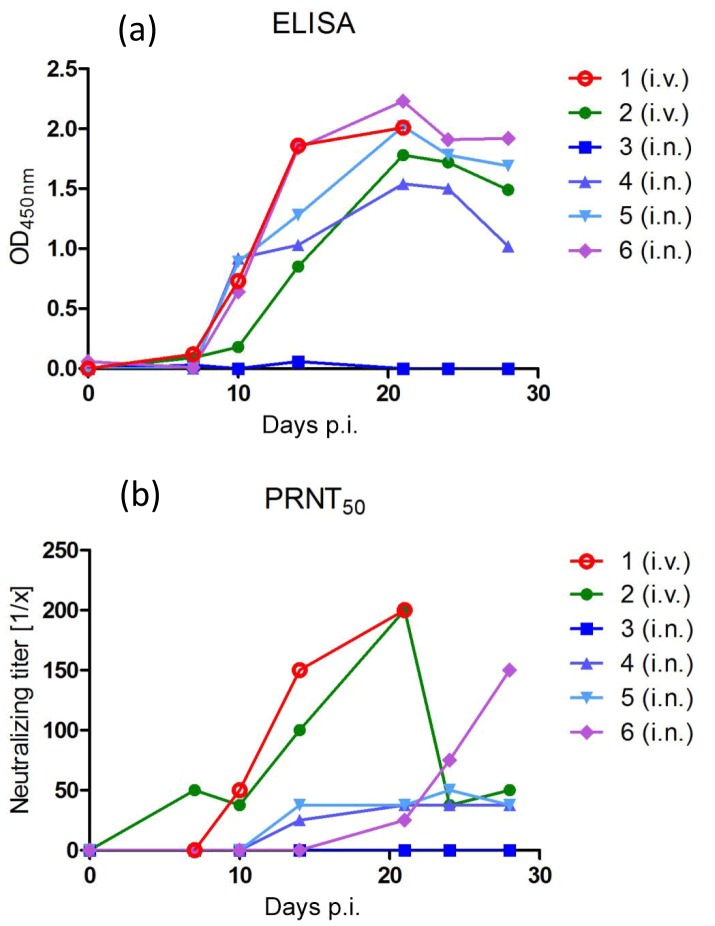
Detection of calpox-virus-specific antibodies in plasma of infected animals. At different points in time post-infection (see Sect. 2.1), whole blood was collected and plasma was prepared. **(a)** Antibodies specifically binding calpox viral proteins were measured by ELISA and are shown as optical densities at 450 nm using plasma diluted 1 : 400. **(b)** Neutralizing antibodies were determined with the plaque reduction neutralization assay (PRNT). The data are expressed as 50 % plaque reduction titers. Animal numbers and the different inoculation routes (i.v., intravenous; i.n., intranasal) are given.

OPXV-specific binding antibodies were detectable by ELISA in plasma of both animals (Fig. 3a). Animal 1 and 2 seroconverted between day 7 and 10, and antibody levels increased until 3 weeks post-infection. As analyzed by PRNT, both intravenously infected animals started to develop neutralizing antibodies against OPXV between day 7 and 10. Maximum titers of 1 : 200 were observed between day 21 and 28 post-infection.

#### 3.1.3 Histologic investigation

**Figure 4:**
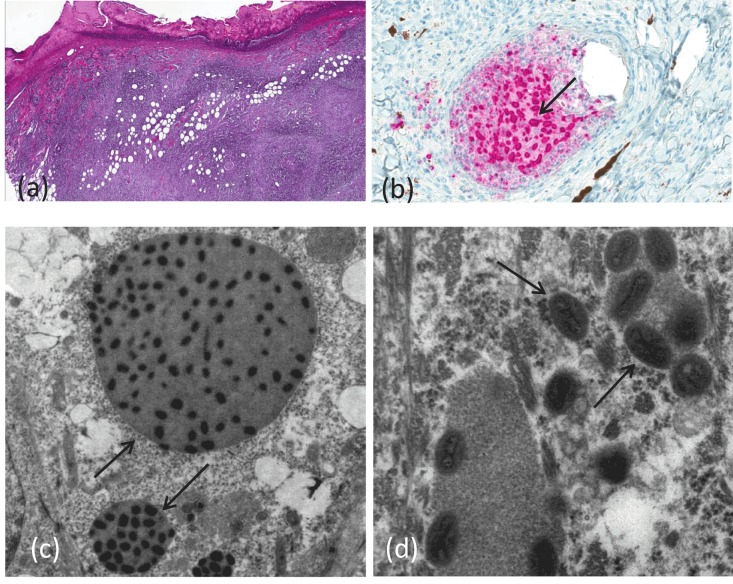
Dermal lesions at time of death (day 21 p.i. after i.v. calpox infection), *Macaca mulatta*, animal no. 1. **(a)** Severe subacute dermatitis with epidermal ulceration covered with serocellular crusts and severe granulomatous inflammation in deeper parts of the dermis (HE stain). **(b)** Inflammation of a sebaceous gland with typical intracytoplasmic Guarnieri bodies (arrow) positive for calpox virus antigen in immunohistochemistry. **(c)** Transmission electron microscopy (TEM) showing multiple intracytoplasmic inclusions in infected epithelial cells which contain mature viral particles (arrows). **(d)** Enveloped viral particles were ovoid to brick shaped, have a pale central zone and a size of 140 × 260 nm (arrows; TEM).

Pathohistological investigation revealed typical poxvirus-induced skin lesions in animal 1. The lesions were characterized by superficial ulceration of the epidermis. The ulcerated parts were covered by a serocellular crust (Fig. 4a). Adjacent parts of the epidermis showed irregular epidermal hyperplasia and signs of acantholysis, acanthosis and syncytia formation of the basal keratinocytes. Numerous large Guarnieri bodies were found in altered epithelial cells. In deeper parts of the dermis, follicular and sebaceous epithelia were also affected, leading to severe chronic granulomatous infection. The inflammatory infiltrate was predominated by lymphocytes and histiocytes and extended deeply into dermis and subcutis. Characteristic intracytoplasmic inclusion bodies were also found in enlarged vacuolated or degenerated cells of sebaceous glands (Fig. 4b). The Guarnieri bodies measured 2–8 µm and were distributed randomly within the altered epithelium. Animal 2 did not show pox-specific alterations.

**Table 2:** Detection of infectious particles in saliva.

	Days p.i.						
Animal no.	4	7	10	14	17	21	24
(route)							
2 (i.v.)	++	++	++	++	++	++	+
3 (i.n.)	-	-	-	-	-	-	-
4 (i.n.)	-	-	-	-	-	-	-
5 (i.n.)	+	n.d.	-	-	-	-	-
6 (i.n.)	+++	n.d.	n.d.	+++	-	-	-

No plaques (-), 1 plaque (+), 2–10 plaques (++), > 10 plaques (+++), not done (n.d.); i.v., intravenous; i.n., intranasal, p.i., post-infection.

#### 3.1.4 Immunohistochemical and ultrastructural findings

Immunohistochemistry and electron microscopy confirmed the presence of calpox-virus-infected cells in the skin of animal 1. In dermal lesions, virus was present in degenerated epithelial cells, dermal macrophages and altered sebaceous glands (Fig. 4b). The virus was not detected in any of the other organs tested. Electron microscopy revealed virus particles with OPXV-like morphology in intracytoplasmic inclusions in epithelial cells. Ultrastructurally, numerous intracytoplasmic inclusion bodies were visible (Fig. 4c), presenting mature viral particles with a size of 140 × 260 nm. The enveloped viral particles were ovoid to brick shaped with a pale central zone, presenting characteristic OPXV-like ultrastructural features (Fig. 4d).

### 3.2 Intranasal inoculation without clinical disease

Only one of the animals infected by the i.n. route showed mild clinical symptoms. This animal (3) developed an enanthema of the pharynx mucosa at day 14 p.i., where three single papules occurred at day 17 and one at the corner of the right eye. All lesions were completely healed by day 21. The other three intranasally infected animals showed neither clinical symptoms nor pathomorphologic alterations related to the calpox virus infection. No viral DNA was detected in blood or tissues of the animals infected intranasally in contrast to the intravenously infected ones (Fig. 2a and b). Nevertheless, infectious virus was sporadically isolated in saliva of animals 5 and 6. With the exception of animal 3, OPXV-specific binding antibodies were detectable by ELISA in plasma of all i.n. inoculated animals (Fig. 3a). All three animals seroconverted between day 7 and 10, and antibody levels increased until 3 weeks post-infection. They also developed neutralizing antibodies that appeared between day 14 and 21 after inoculation. Titers were generally lower in these animals compared to the i.v. infected ones. Interestingly, in one of these three animals (animal 6), neutralizing titers increased continuously until the end of the follow-up (Fig. 3b).

## 4 Discussion

We previously showed that experimental low-dose intranasal infection of common marmosets with the calpox virus results in fatal disease. The calpox virus belongs to the *Cowpox virus* species and was isolated from *Callithrix jacchus* during a natural outbreak of the disease in a private New World monkey husbandry (Mätz-Rensing et al., 2006). High doses of the virus experimentally applied to marmosets reproducibly led to death within 4 to 7 days. Even the intranasal application of as low as 50 PFU of calpox virus to the New World monkeys was infectious in 40 % of the animals and resulted in viremia and fatal outcome (Kramski et al., 2010). The route and dose used for viral inoculation of this monkey species mimics the natural transmission of smallpox, thus representing a suitable model to study pathogenesis and to evaluate new vaccines and therapeutics against orthopoxvirus infection (Kramski et al., 2010; Mätz-Rensing et al., 2012). However, this animal model has some limitations due to the lack of species-specific or cross-reactive reagents, particularly for the analysis of innate immune responses which hamper certain experimental approaches. The aim of the present study was to test whether the results from the marmoset studies can be recapitulated in rhesus macaques, a nonhuman primate species that has been used in many different research fields for decades.

### 4.1 Rhesus macaques are less susceptible to calpox virus exposure

The results of the study suggest that New World monkeys, i.e., common marmosets, are more susceptible to the calpox virus than rhesus monkeys. High levels of viral replication were observed in blood accompanied by the appearance of classical pox lesions of varying degree only when this Old World monkey species was given high doses in the range of 10${}^{6}$ PFU of infectious virus intravenously. A comparable dose applied to marmosets reproducibly led to death within 4 to 7 days (Kramski et al., 2010). A dose of 8.3 × 10${}^{3}$ PFU reliably led to infection and 100 % mortality in marmosets (Mätz-Rensing et al., 2012), while an approximately 100-fold higher dose of calpox virus inoculated intranasally in rhesus monkeys compared to that used in marmosets was not sufficient to induce viremia, let alone conspicuous clinical alterations. Nonetheless, seroconversion in three out of four rhesus monkeys inoculated intranasally and the detection of infectious virus in saliva of two of the three seroconverted animals suggest local low-level replication, presumably at the portal of virus entry, which could be the nasal epithelium. Those findings can be defined as subclinical infection.

### 4.2 Symptomatic infection requires high virus doses

In contrast to marmosets, a very high dose of calpox virus is needed to infect rhesus macaques. Nevertheless, the clinical symptoms observed in the rhesus macaques infected intravenously were typical of an orthopoxvirus infection. Both animals developed viremia followed by characteristic skin lesions. Whereas the lesions in animal 1 showed similarities with the confluent ordinary smallpox type with a higher fatality rate, lesions in animal 2 could be compared with a mild discrete ordinary type of smallpox. In both animals, the pustules on the face, arms and extremities were numerous and rather sparse on the trunk. Lesions were all at the same stage; those that appeared earliest on face and upper extremities were more mature than those that appeared later on other parts of the body. Lesions were also present on soles and palms. Except on soles and palms, umbilication was a common feature of the skin lesions similar to smallpox disease. Histological analyses demonstrated that cells of the sebaceous glands were highly susceptible, which is typical of the infection with other cowpox viruses or smallpox.

Furthermore, the older age of the monkeys in this study should be considered. Upon necropsy, all animals presented with minor chronic diseases, such as pulmonary acariasis induced by *Pneumonyssus semicola* or endometriosis (see Table 1), which often represent clinically inapparent lesions in older animals. This could lead to the assumption that these clinically inapparent diseases as well as the old age of the monkeys might have affected their ability to resist the viruses, making them more susceptible to the administered calpox virus. In humans, children and elderly people seem to be more vulnerable to orthopoxvirus infections than middle-aged people (Fenner et al., 1989). However, this does not seem to be the case with the calpox virus in rhesus macaques. Apparently, old age does not seem to increase the susceptibility to this virus. Intranasally infected rhesus monkeys remained healthy, and animals infected by the i.v. route developed relatively moderate symptoms in relation to the high viral dose that was inoculated. This confirms that the calpox virus is less pathogenic to macaques compared to marmosets and leads to the question of species-specific pathogenicity that seems to be a phenomenon of CPXV.

### 4.3 Species-specific pathogenicity and host range phenomena

Despite high genetic homology, OPXV shows great differences in host range. On the one hand, VARV, which is restricted to humans, has no known natural reservoir and is less pathogenic to nonhuman primates. On the other hand, CPXV with a known reservoir in rodents has a broad host range and is responsible for lethal CPXV infections in different animal species and even humans (Essbauer et al., 2010; Eis-Hübinger et al., 1990). The combination of the CPXV strain and host seems to play an important role in pathogenesis and disease outcome. A CPXV infection in humans or cats usually leads to a self-limiting local infection, while the same virus strain induces death in susceptible animal species as described for banded mongooses and jaguarundis (Kurth et al., 2009). Similar observations were made for other CPXV–host combinations (Kurth et al., 2008). It could be shown that the calpox virus also belonging to the CPXV species led to highly reproducible lethal disease in marmosets but was less pathogenic in mice (unpublished data). The results of the present study demonstrated a lower pathogenicity of the same virus for Old World monkeys and underline the great influence of virus–host interrelationships for disease outcome. The molecular factors which determine the host range and host-specific pathogenicity are not very well understood yet. Taking all these facts into account, it could be assumed that the cowpox virus, which represents an Old World virus, has a higher virulence for New World species such as marmosets, jaguarundis or prairie dogs.

### 4.4 Susceptibility of macaques to other CPXV strains

The observation that the calpox virus is less pathogenic to rhesus macaques led to the question whether Old World monkeys are susceptible to CPXV in general. During a natural cowpox outbreak in a sanctuary for exotic animals in the Netherlands, several animals of different macaque species developed neutralizing serum antibody titers, indicating an exposure to the virus, but only three of them developed mild clinical symptoms. Experience of this outbreak suggests that macaques are susceptible to cowpox virus but that the clinical outcome is less severe than in New World monkeys (Martina et al., 2006; Mätz-Rensing et al., 2006). There is evidence that cynomolgus and rhesus macaques are susceptible to CPXV Brighton Red (CPXV-BR). Recently, 14 cynomolgus macaques were inoculated intravenously with different doses of CPXV Brighton Red (5 × 10${}^{4}$ - 5 × 10${}^{7}$ - PFU; Johnson et al., 2011b). A total of 9 out of 14 animals developed typical pox-like skin lesions. Further findings included hemorrhages in a variety of organs, indicating a hemorrhagic course of disease. Infection was uniformly lethal within 12 days post-inoculation. The researchers concluded that this animal model may serve as a model for hemorrhagic smallpox, which is more feasible than the VARV model (Jahrling et al., 2004). The same researchers adapted a method of the intrabronchial and intra-alveolar infection with CPXV-BR to mimic the natural route of infection more closely. Intrabronchial inoculation and small-particle aerosol inoculation of cowpox BR led to a severe respiratory disease in both macaque species (Smith et al., 2012; Johnson et al., 2015). Macaques inoculated by aerosol developed severe bronchointerstitial necrotizing pneumonia, but skin lesions were not observed and the viral dissemination was limited. This shows that the route of infection strongly influences disease progression.

## 5 Conclusion

Rhesus macaques are less susceptible to calpox virus exposure. Symptomatic infection requires high virus doses and depends on the route of application. Therefore, the rhesus monkey calpox model is not suited for calpox virus research and is of limited use in further intervention studies against OPXV.

## Data Availability

All relevant data are presented in the paper. Please contact the corresponding author for further details.
